# Foraging movements of breeding Kelp Gulls in South Africa

**DOI:** 10.1186/s40462-020-00221-x

**Published:** 2020-09-03

**Authors:** Katharina Reusch, Nicolás Suárez, Peter G. Ryan, Lorien Pichegru

**Affiliations:** 1 Institute for Coastal and Marine Research, Department of Zoology, Nelson Mandela University, PO Box 77000, Port Elizabeth, South Africa; 2 Centro para el Estudio de Sistemas Marinos, CCT CENPAT-CONICET, Puerto Madryn, Chubut Argentina; 3 FitzPatrick Institute of African Ornithology, DST-NRF Centre of Excellence, University of Cape Town, Rondebosch, Cape Town, 7701 South Africa

**Keywords:** Seabird ecology, *Larus dominicanus*, Bio-logging, Anthropogenic food

## Abstract

**Background:**

Kelp Gulls *Larus dominicanus* are one of the most abundant gulls in the Southern Hemisphere and can play an important role in their ecosystem. Understanding their foraging ecology is therefore important, especially in the context of anthropogenic changes of the environment. Over 35,000 Kelp Gulls breed in South Africa but little is known about their habitat use. It has been hypothesised that foraging mainly occurs in natural habitats while provisioning chicks to ensure high quality food, but knowledge on their foraging ecology during the incubation period remains poor.

**Methods:**

We tracked incubating Kelp Gulls from six colonies distributed along the coast of South Africa, varying in their distance to urban areas and landfills, and compared foraging trip patterns and habitat selection between colonies.

**Results:**

Gulls from west coast colonies, generally located further from landfills than the other studied colonies, travelled farther from their breeding sites (11.7 ± 9.9–17.8 ± 21.7 km, *n* = 3 colonies) than birds from Cape Town and south and east coast colonies (1.7 ± 0.8–3.1 ± 3.7 km, *n* = 3) with birds travelling farthest when foraging at sea. Gulls from all colonies spent more time foraging in marine, coastal, and natural terrestrial environments than scavenging in strongly modified habitats while incubating.

**Conclusions:**

Our results suggest that Kelp Gulls in South Africa are able to exploit various resources from different foraging habitats, regardless of colony location and seem to rely less on anthropogenic habitats than expected.

## Background

Humans are having increasingly profound impacts on the environment through a myriad of activities including urbanization, contributing towards global changes [1]. Some species show greater tolerance towards anthropogenic changes than others, for example by being less specialised in terms of habitat or diet and can benefit from altered conditions. Such species are considered ‘winners’ of global changes (e.g. [2]). By comparison, more specialised species with more sensitive requirements tend to be limited in their capacities to adapt to changes, and often experience population and range decreases as a result of global changes (e.g. [2–4]).

Seabirds are particularly threatened by global changes with 28% of seabirds being categorised as either critically endangered, endangered or vulnerable [5]. As seabirds use both marine and terrestrial habitats ([5, 6]), threats include overfishing inducing depletion of their prey, bycatch in fisheries, pollution, introduced species in their breeding sites, anthropogenic disturbance, and habitat loss [5]. Populations of specialist feeders in particular tend to have declined (e.g. [7–9]) due to major ecological changes [10], as well as competition with fisheries [11]. By contrast, opportunistic and scavenging species are generally advantaged and some of their populations are growing exponentially in several parts of the world (e.g. [12–15]). Opportunistic seabirds that are able to switch to alternative food sources can become a problem for other seabirds through competition for prey or direct predation, when extensively used food sources, like fishery discards [16] or offal [17] are reduced.

Many large gulls (*Larus* spp.) are opportunistic foragers, able to exploit a wide variety of food sources ranging from marine to intertidal, terrestrial, or anthropogenic (e.g. [18, 19]). Their ability to forage on human-derived food, such as fishery waste and open refuse tips, as well as cessation of population control measures has led to an increase in population numbers for many species since the 1970s [13, 20]. Food derived from anthropogenic sources can be more predictable and easily accessible [21] than food derived from e.g. the marine environment, which can often be patchily distributed [22]. Even though many gull species feed opportunistically throughout the year, there seems to be a switch in diet during the chick-rearing period to more natural prey, e.g. fish [23, 24]. This selective behaviour might be related to the fact that natural prey has a higher nutritional value and is more easily handled by chicks [23].

The Kelp Gull *Larus dominicanus* is distributed in coastal areas and on islands at mid-to high-latitudes throughout much of the southern hemisphere [25]. Kelp Gull populations are generally increasing, with a global estimate of 3.3 to 4.3 million individuals [25]. Population increases in both South America [26] and South Africa [27] have been attributed to increased feeding opportunities mostly from anthropogenic sources [13, 28, 29]. Kelp Gulls are opportunistic feeders that forage on a wide variety of natural prey as well as food derived from human activities [30–33]. In South Africa, the breeding population is estimated at about 17,500 pairs [27] and they are known to feed on invertebrates, fish, insects, berries, frogs, snakes, small mammals and carcasses of birds and seals as well as seabirds’ eggs and chicks including conspecifics [34]. They also scavenge from rubbish dumps, fishing harbours and croplands [34].

Knowledge of Kelp Gull foraging ecology is limited in South Africa and information exists mostly on abundance (e.g. [27, 35]) and distribution patterns (e.g. [28, 36]), as well as their general diet (e.g. [30, 34]). As Kelp Gulls can have an important role in their ecosystem due to their abundance it is important to understand their foraging ecology. Kelp Gulls are generalists and can be predators of other seabirds, so variations in the availability of their main food sources could affect other seabirds. In addition, a decrease in the availability of supplementary food sources could cause population declines, as seen in other gull species [37, 38]. Gulls tend to switch to a more natural diet during chick-rearing (e.g. [23, 24]), therefore the incubation period may provide the opportunity to get a more comprehensive insight into the wider range of foraging habitats exploited, and to identify the relative importance of anthropogenic resources for adult Kelp Gulls. In this study we investigated the foraging behaviour of Kelp Gulls breeding at six colonies in South Africa, varying in their proximity to urban areas and landfills. We deployed GPS loggers on incubating adults to explore: 1) whether colony location in relation to anthropogenic areas influenced foraging effort, and 2) whether foraging habitat choice (e.g. oceanic, terrestrial or anthropogenic, like landfills) differed among colonies. We expected birds from colonies located closer to urban areas or landfills to have reduced foraging effort and to rely more on anthropogenic food.

## Methods

**Fig. 1:**
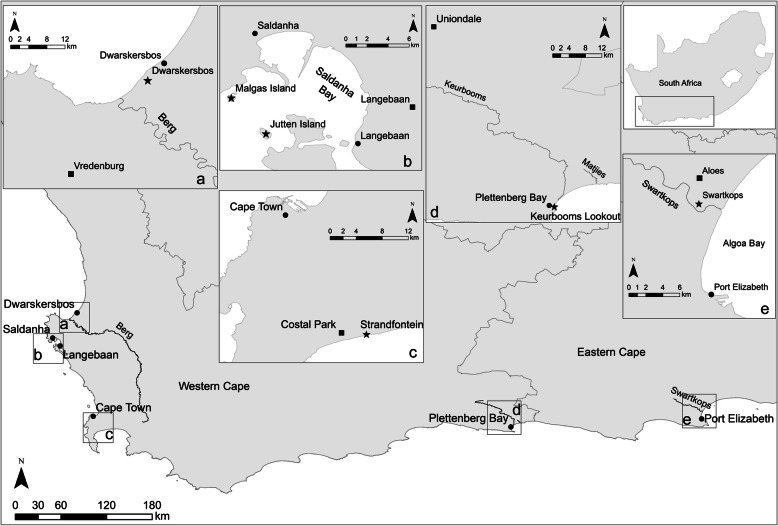
Map of the study areas showing the locations of the six Kelp Gull colonies in South Africa (stars), closest cities (circles), and closest landfills (squares)

The foraging behaviour of incubating adult Kelp Gulls was investigated at six colonies, three on the west coast, one within Cape Town, one on the south coast and one on the east coast of South Africa (Fig. 1).

The Dwarskersbos colony (DW; 32°43′S, 18°12′E) is located on the west coast of South Africa in a salt works 4 km south of Dwarskersbos. This small coastal village with 670 inhabitants is 25 km from the closest landfill. The colony had some 1200 Kelp Gull breeding pairs in 2018 (L. Upfold, pers. comm.).

Malgas Island (MA; 33°03′S, 17°55′E) and Jutten Island (JU; 33°05′S, 17°57′E) are small islands in the West Coast National Park in the mouth of Saldanha Bay on the west coast of South Africa. Malgas Island had 113 breeding pairs of Kelp Gulls in 2018 (B. Dyer, pers. comm.). The 8.3 ha island lying 850 m offshore, is home to colonies of Cape Gannets *Morus capensis*, Bank *Phalacrocorax neglectus*, Cape *Phalacrocorax capensis*, and Crowned Cormorants *Microcarbo coronatus*. The closest towns are Saldanha and Langebaan, with 28,000 and 8000 inhabitants, respectively, with the closest landfill 15 km from the colony.

Jutten Island (JU; 33°05′S, 17°57′E; 46 ha), 3.7 km southeast of Malgas Island, had ca 1200 Kelp Gull breeding pairs in 2018 (B. Dyer, pers. comm.) and is roughly 12 km away from the closest landfill. Other seabirds breeding on the island include Crowned, Cape and Bank Cormorants.

The Strandfontein colony (ST; 34°05.4′S, 18° 32.1′E) is located within the city of Cape Town with 3.4 million inhabitants. The colony is located in sandy dunes between Strandfontein sewage works and False Bay and is only 3 km from the large Coastal Park landfill site. It had ca 1060 breeding pairs in 2018 (B. Dyer, pers. comm.).

The Keurbooms Lookout Beach colony (KE; 34°03′S, 23°22′E) is located on a sandbank at the western side of the Keurbooms Estuary, in Plettenberg Bay on the south coast. The colony is situated 1 km from Plettenberg Bay with 32,000 inhabitants and 51 km from the closest landfill. The colony is the smallest colony sampled with 100 Kelp Gull breeding pairs (pers. obs. 2017), but lies adjacent to a much larger colony of some 1300 pairs on the Keurbooms Peninsula [27].

Finally, the Swartkops Estuary colony (SW; 33°51′S, 25°34′E) is located in Port Elizabeth, a city of approximately 1.2 million inhabitants, on the Swartkops Estuary on the east coast. This estuary is generally in poor condition due to various anthropogenic sources of pollution, including sewage discharges (Adams et al., 2019). The colony is 3 km from the nearest landfill and has some 500 Kelp Gull breeding pairs (P. Martin 2019, pers. comm.).

### GPS deployment and analyses

Miniaturized GPS loggers (CatTrack/I-gotU 44.5 × 28.5 × 13 mm, Perthold Engineering LLC/Mobile Action) were deployed on one incubating Kelp Gull per nest at 6 colonies in October–November 2017 and at four colonies in October 2018 (see Additional file 2). Birds were captured initially with a walk-in trap and recaptured with a noose placed over the nest after a period of 24 to 96 h. Upon capture, all gulls were weighed and GPS loggers were taped to the back feathers with Tesa tape®, which causes limited damage to the plumage [39]. GPS loggers weighed ~ 20 g, representing ≤2.7% of the birds’ body weight (730–1200 g, [40]), and were programmed to record a position every 30 s in 2017 and every 3 min in 2018. This longer interval was set to increase the battery life of the loggers and record additional foraging trips. Handling time was ~ 5 min for GPS deployment. Upon release all birds were marked with non-toxic animal dye to allow identification. Upon recapture, GPS loggers were removed, gulls were re-weighed and measured: head length, bill length and depth, and tarsus length to the nearest 0.1 mm with Vernier callipers, and wing length (flattened chord) to the nearest 1 mm using a stopped wing ruler. Due to additional samples collected for another study, handling time after recapture was ~ 10–12 min. To test whether GPS deployment and handling may have a detrimental effect on the birds, we compared their weight prior to GPS deployment and after recapture with a paired t-test. The differences were not significant (*n* = 69; t = − 70; *p* > 0.05), implying that the effect of our study was negligible on the birds. In addition, we observed that most birds stayed on the colony after release and started incubating within 10 min.

GPS data were uploaded into ArcMap 10.5.1 (Esri, 2018) to identify foraging trips. We only considered trips away from the colony lasting > 10 min as foraging trips < 10 min were mostly within 1 km of the breeding site and were most likely for comfort behaviours (i.e. bathing, roosting; see Additional file 1). All GPS data were filtered for erroneous GPS locations following [41], based on a maximum flying speed of 70 km h^− 1^ [42]. As the sampling interval differed between GPS tracks in 2017 and 2018, tracks were interpolated to a common interval of 3 min using the function redisltraj in the R package adehabitatLT [43] allowing comparisons between years. For each trip, maximum distance from the colony (greatest distance from last point on colony), path length (sum of distance between all consecutive GPS locations) and trip duration (time between last and first location on the colony before and after a trip) were calculated.

### Habitat analysis

In order to identify foraging areas, we used an Expectation-Maximization Binary Clustering (EMbC) algorithm for behavioural annotation using the R package EMbC [44]. This algorithm used turning angle and speed between successive GPS locations to assign each location to one of four behavioural categories: low velocity and low turns (LL), high velocity and low turns (HL), low velocity and high turns (LH), and high velocity and high turns (HH) [44]. We considered that an animal was flying when the velocity was high and the turning angle low (HL) and potentially foraging in all other three categories (LL, LH, HH). Foraging locations were then uploaded into ArcMap 10.5.1 and an Imagery Basemap was used to associate them with a defined foraging habitat. Foraging habitats were categorised as follows: oceanic, coastal, terrestrial natural and terrestrial anthropogenic. Oceanic habitat was defined as any point in the marine environment > 60 m from the shore. Coastal habitats included the shore (beach and up to 60 m from the shoreline). Terrestrial natural habitats consisted of unmodified terrestrial habitats such as nature reserves, and terrestrial anthropogenic habitats were defined as transformed areas (e.g. artificial water bodies, parks), urban areas, landfills, and agricultural fields.

### Statistical analysis

All statistical analyses were carried out in R (version 3.5.2 [45];). To compare foraging trip parameters (maximum distance from the colony, trip duration, path length) between colonies and years, we fitted models using the lmer function from the lmerTest package [46]. Trip parameters were log transformed to obtain normality and homoscedasticity and set as the response variable, while year and colony were the explanatory variables, with bird ID as a random factor to account for multiple trips per bird. The MuMIn package [47] was used for averaging the different models and selecting the best fit model based on the Akaike Information Criterion (AIC). We performed post hoc Tukey tests on the explanatory variable of each model to allow pair-wise comparisons using the multcomp package [48].

We then compared the use of different foraging habitats (oceanic, coastal, terrestrial natural, terrestrial anthropogenic) between colonies and whether foraging parameters were influenced by foraging habitat choice. A correlation matrix was used to assess the level of correlation between trip parameters in order to avoid any effect of collinearity in our results. The “Pearson” method with the Hmisc package [49] was used to obtain significance levels for correlations. As trip parameters were strongly correlated (r values between 0.6–0.99 and *p* values < 0.001), trip duration and path length were removed from habitat choice analysis. In order to maximise the accuracy of the model, maximum distance was used, as it showed more significant differences between colonies. Only the dominant habitat (i.e. that most visited during a foraging trip, > 50% of foraging time) was considered in each trip. Habitats were combined when an individual spent an equal amount of time in more than one habitat. We used a conditional inference tree with the function ctree of the party package [50] to estimate what influenced habitat choice and used the type of habitat as response variable and maximum distance and colony as explanatory variables. We used the default setting to build the tree and set statistical significance at *p* ≤ 0.05. To estimate model accuracy, we set the seed at 1234 and divided the data set into a training (70% of data) and testing data set (30% of data). Model accuracy was obtained by calculating the misclassification error rate on the testing data set following [51].

## Results

Of the 85 incubating Kelp Gulls equipped with GPS loggers, 75 were recaptured. All 10 birds that eluded recapture were observed alive. Of the 75 recaptured birds, one GPS logger was damaged and data could not be retrieved, two loggers did not record data in a consistent way and one bird had lost the GPS. In addition, in 2017 three birds did not leave their colony. As a result, data were collected from 68 birds, which completed 316 foraging trips (see Additional file 2).

### Trip parameters

**Fig. 2:**
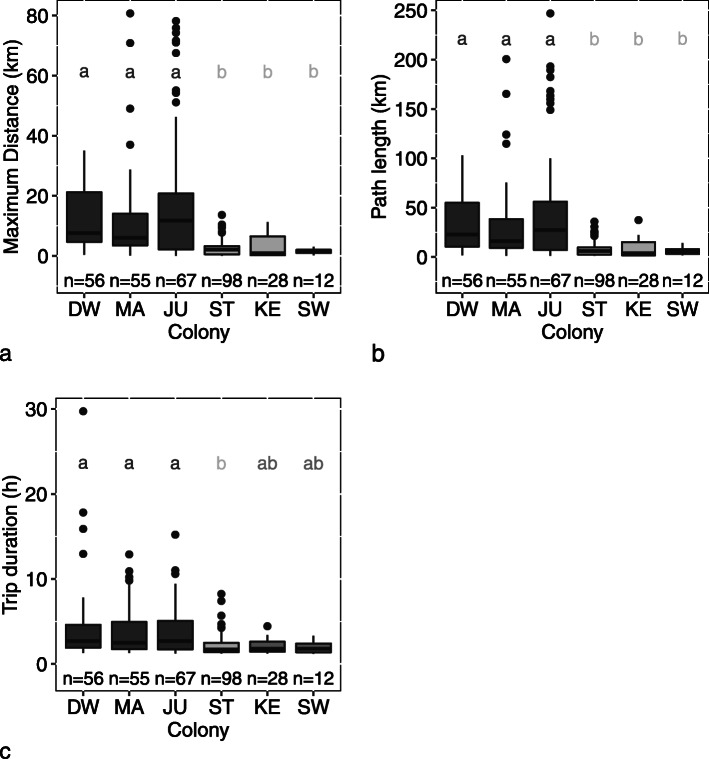
Boxplots representing foraging trip parameters a) maximum distance (km), b) path length (km), and c) trip duration (h) of incubating Kelp Gulls from six South African colonies (DW = Dwarskersbos, MA = Malgas Island, JU = Jutten Island, ST = Strandfontein, KE = Keurbooms, SW = Swartkops) in 2017–2018. The boxplots show the median values (band inside box), the 25th and 75th percentiles (box), the smallest and highest value within 1.5 times interquartile range (whiskers) and outliers (dots). N represents the number of trips per colony and boxes and letters above with different shades of grey are significantly different from Tukey test results

Foraging trip parameters did not vary between years (lmer, *p* > 0.05), allowing data from both years to be pooled for comparisons between colonies. Birds from Jutten Island foraged farthest from their colony, with an average maximum distance ± SD of 17.8 ± 21.7 km (Range = 0.03–78.2 km; *n* = 56), compared to averages varying between 1.7 ± 0.8 and 11.8 ± 15.9 km (Range = 0.07–80.7 km; *n* = 206) at the five other colonies (Fig. 2). Gulls from Cape Town and south and east coast colonies, Strandfontein, Keurbooms, and Swartkops, all foraged close to their colony, i.e. 1.7 ± 0.8 to 3.1 ± 3.7 km (Range = 0.07–13.66; *n* = 138) from their breeding sites, with birds from Swartkops travelling the shortest distances. Maximum distances and path lengths varied significantly between colonies (lmer, *p* < 0.001), with birds from west coast colonies, Dwarskersbos, Malgas and Jutten travelling farther and with longer path lengths than birds from the three other colonies. Trip durations also differed between colonies with trips from Strandfontein being significantly shorter than trips from the west coast colonies (lmer, *p* < 0.001; Table 1). Trip durations ranged from a minimum of 12.6 min at Swartkops up to a maximum of 28.7 h in Dwarskersbos and foraging distances from 30 m from the colony at Jutten, up to 80 km at Malgas.

**Table 1:** Summary statistics of colony influence on maximum distance (km), path length (km), and duration (h)

Model	Fixed factors	Model estimates ± SE	AICc	DF	R^2^m	R^2^c
Log (Max distance) ~ Intercept			1152.41	3		
Log (Max distance) ~ Colony	Intercept DW	1.94 ± 0.21	1108.95	8	0.26	0.31
	Colony MA	−0.17 ± 0.30				
	Colony JU	0.07 ± 0.28				
	Colony ST	−1.66 ± 0.27				
	Colony KE	−1.79 ± 0.36				
	Colony SW	−1.58 ± 0.48				
Log (Path length) ~ Intercept			1161.91			
Log (Path length) ~ Colony	Intercept DW	2.86 ± 0.20	1118.01	5	0.25	0.28
	Colony MA	−0.16 ± 0.29				
	Colony JU	0.05 ± 0.27				
	Colony ST	−1.63 ± 0.26				
	Colony KE	−1.78 ± 0.35				
	Colony SW	−1.48 ± 0.47				
Log (Trip duration) ~ Intercept			906.09			
Log (Trip duration) ~ Colony	Intercept DW	0.55 ± 0.15	888.43	5	0.14	0.21
	Colony MA	−0.03 ± 0.22				
	Colony JU	−0.01 ± 0.20				
	Colony ST	−0.80 ± 0.20				
	Colony KE	−0.71 ± 0.27				
	Colony SW	−0.88 ± 0.35				

We used linear mixed-effect models with colony as fixed factors and bird ID as random intercept. All response variables were log transformed. Intercept DW is the intercept and the estimate for the Colony DW. Model estimates and standard errors are shown for the six colonies. We provided the marginal R^2^ which represents the variance explained by the fixed factors alone, and the conditional R^2^ which describes the variance explained by both the fixed and random factors. Colonies *DW* Dwarskersbos, *MA* Malgas Island, *JU* Jutten Island, *ST* Strandfontein, *KE* Keurbooms, *SW* Swartkops

### Habitat analysis

**Fig. 3:**
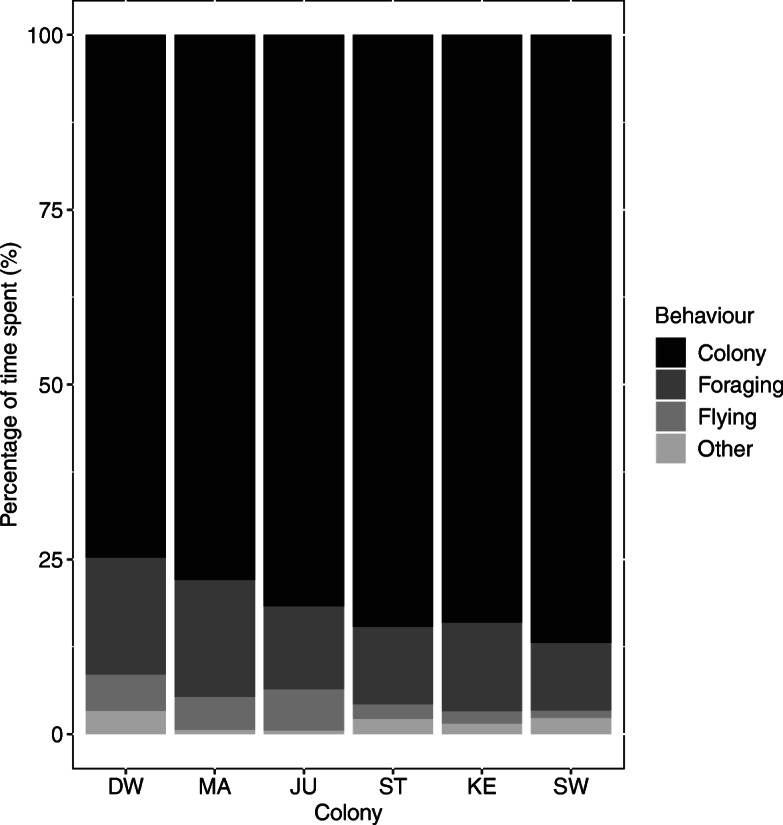
Percentage of time spent by incubating Kelp Gulls at the colony, foraging, flying, or other (i.e. trips < 10 min outside the breeding colony) for each of the six South African colonies (DW = Dwarskersbos, MA = Malgas Island, JU = Jutten Island, ST = Strandfontein, KE = Keurbooms, SW = Swartkops)

Birds from all six colonies spent 75–87% of their time within the colony, 10–17% foraging and 1–6% flying (Fig. 3). Trips < 10 min outside the breeding colony are represented as “other” (0.5 to 3% of time). Overall, birds from Dwarskersbos and Malgas Island spent the longest time away from the colony foraging (17%), while birds from Swartkops foraged for only 10% of the time.

**Fig. 4:**
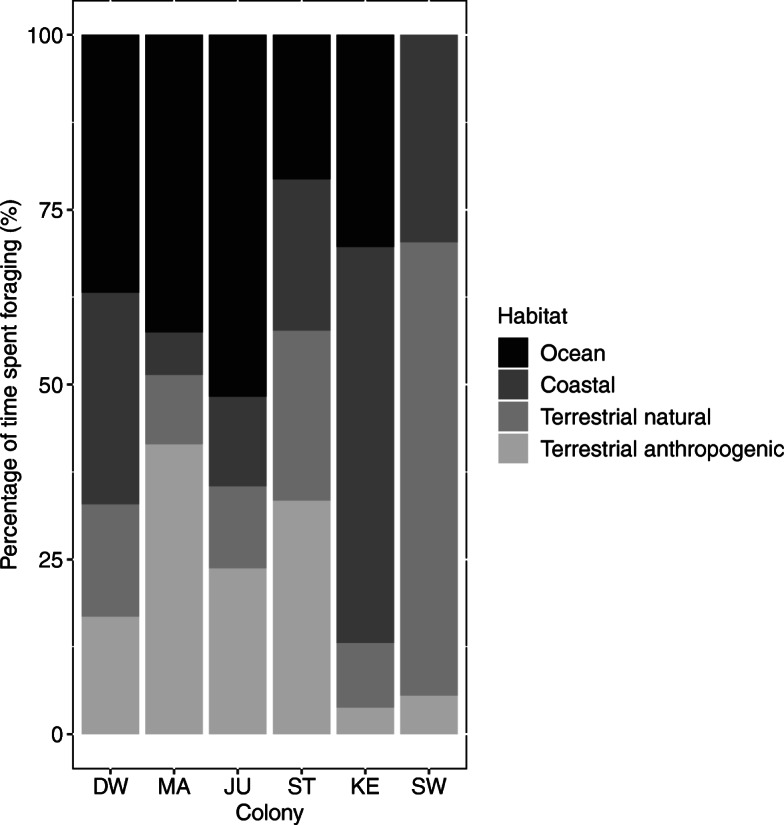
Percentage of time spent by incubating Kelp Gulls in each of the four foraging habitats for each of the six studied colonies in South Africa (DW = Dwarskersbos, MA = Malgas Island, JU = Jutten Island, ST = Strandfontein, KE = Keurbooms, SW = Swartkops)

**Fig. 5:**
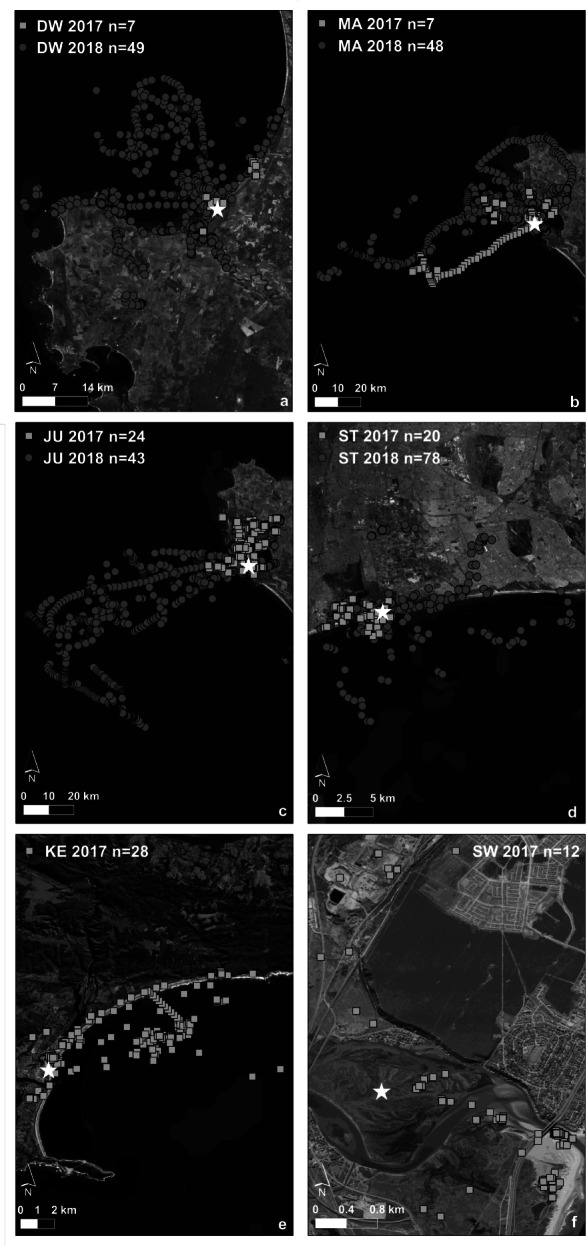
**a**-**f**: Foraging locations of incubating Kelp Gulls for each of the six colonies (DW = Dwarskersbos, MA = Malgas Island, JU = Jutten Island, ST = Strandfontein, KE = Keurbooms, SW = Swartkops). The colony is represented by a white star, foraging locations from 2017 are shown in light grey squares and from 2018 in dark grey dots, respectively

Foraging habitats, as identified by the EMbC, varied widely across colonies (Fig. 4, see Additional file 3). Gulls from Dwarskersbos, Malgas, and Jutten Island, i.e. the west coast colonies, mostly foraged in oceanic habitats (37–52% of their foraging time), whereas birds from Keurbooms foraged mainly in coastal habitats (57%) and from Swartkops in terrestrial natural areas (65%; Fig. 5). Birds from Strandfontein showed more diverse habitat choices, foraging in oceanic, coastal, terrestrial natural and terrestrial anthropogenic habitats. Birds from all colonies fed to some extent in terrestrial anthropogenic areas (4–41% of their time), with birds from Malgas Island (41%) and Strandfontein (33%) spending the highest amount of time. Birds from Malgas spent more time on artificial water bodies (19%) and agricultural fields (16%) compared to birds from Strandfontein which frequented the sewage works (15%) and nearby landfill (15%). However, much of this time at wetlands could be spent roosting, bathing, or in other comfort behaviours, which could indicate that time spend foraging in these areas was overestimated.

**Fig. 6:**
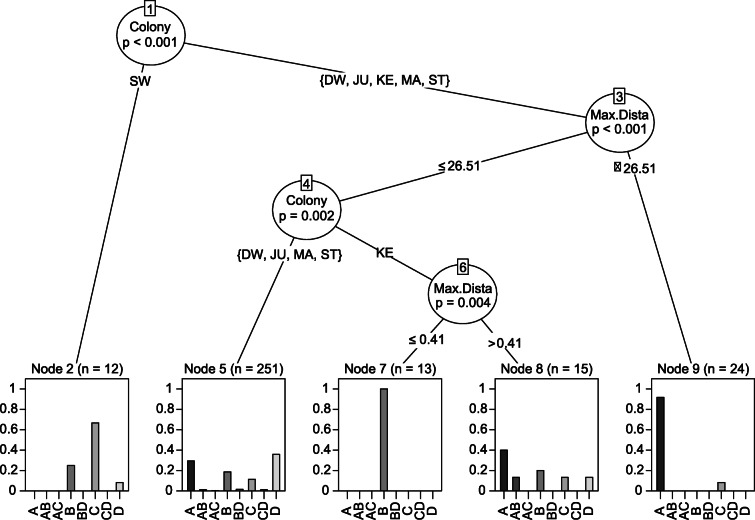
Conditional inference tree for the effect of colony and maximum distance on dominant habitat (A = Ocean, B = Coastal, C = Terrestrial natural, D = Terrestrial anthropogenic, AB, AC, BD, and CD are combinations of the major categories). Each oval contains one of the two explanatory variables, colony or maximum distance. Following the branches lead to the partitions of the variables based on a significance value of *p* ≤ 0.05. The value above each leaf represents the total number of observations that fall within the node. Histograms show the probability of dominant habitat. Colonies DW = Dwarskersbos, MA = Malgas Island, JU = Jutten Island, ST = Strandfontein, KE = Keurbooms, SW = Swartkops

Results from the conditional inference tree showed that foraging habitat choice was significantly influenced by colony (*p* < 0.001; Fig. 6) and maximum foraging distance (*p* < 0.01; Fig. 6), which explained 47% of the variance in habitat choice. The first split (Node 1) showed a significant difference between birds from Swartkops and the other colonies, as the former foraged predominantly in terrestrial natural habitats. The next split showed that birds from all other five colonies were more likely foraging in the marine environment (A, Node 9) when trips were farther from their respective colonies (> 26.51 km; Node 3). The following split revealed different foraging behaviours between colonies again, with Keurbooms colony foraging only in coastal areas (B, Node 7), when foraging ≤0.41 km from their colony, and in the marine environment when foraging further away (Node 8). Finally, birds from Dwarskersbos, Malgas, Jutten and Strandfontein were dividing their time more equally among the four habitats when foraging closer to the colony (Node 5).

## Discussion

Our study showed high foraging flexibility of incubating Kelp Gulls in South Africa. Their foraging range varied from 30 m up to 80 km from the colonies, with colony means ranging between 1.7 ± 0.8 and 17.8 ± 21.7 km, which was generally shorter than other gull species. For example, incubating Ring-billed Gulls *Larus delawarensis* in Canada had an average foraging range of 30.2 ± 23.8 km from their colony, even though they were also breeding close to urban areas [52]. Similarly, incubating Kelp Gulls tracked in Argentina travelled on average 19.6 ± 24.4 km from their colony, while their breeding site was located within 4.5 km of a landfill and birds feeding on the refuse dump made relatively shorter trips (< 7 km) [53]. Kelp Gulls from South Africa made on average shorter trips close to their respective colonies compared to other species, which suggest that they are able to exploit a variety of food sources closer to their breeding sites than other gulls.

In addition, most of their foraging time was spent in natural environments (ocean, coastal, or natural terrestrial), and Kelp Gulls from all South African colonies seemed to rely less on anthropogenic food than expected, even when their colony was located close to urban areas or landfills. These results are in contrast with the foraging ecology of Kelp Gulls in Argentina [53] or Lesser Black-backed Gulls nesting in urban areas in Bristol, UK [54]. In Argentina gulls only spend 10% of their time in the marine environment and often foraged at a landfill site close to the colony (75%), mainly on fishery waste disposed there from recreational activities [53], whereas Lesser Black-backed gulls spent most of their time in anthropogenic habitats and, even though located close to the coast, seldom foraged at sea [54]. The observed difference between urban nesting gulls might reflect differences in prey profitability or availability in their respective environments, with the marine and coastal environments in South Africa seemingly more profitable for gulls. Indeed, foraging profitability can influence foraging habitat choice [52], which in turn will influence foraging distance from the colony [52, 55].

When feeding in the marine environment, Kelp Gulls in South Africa travelled farther away from their colonies, suggesting that foraging at sea may require higher effort but might be balanced as the energetic gain from an oceanic diet is generally higher than food derived from e.g. intertidal areas [56, 57]. It is also possible that farther trips offshore might represent scavenging on fishery discard from trawlers, which Kelp Gulls are known to take advantage of [30, 53]. A higher calorific diet during breeding can lead to a better body condition, which is important for an increased breeding success [58]. By contrast, gulls might chose to forage in more natural areas close to the colony, reducing the energy costs associated with moving to the feeding area [59], which in turn might allow higher nest attendance [58]. As our birds were incubating, i.e. with low energy demands [60], they might choose a “risk averse” feeding strategy [23] as anthropogenic areas such as landfills can be highly competitive [61].

We must bear in mind that our model on habitat choice explained only 47% of the variance using distance travelled and colonies as explanatory variables. The remaining variance might be explained by variables not measured in this study such as weather or energy expenditure [59, 62]. Nevertheless, the results obtained in this study show the high trophic plasticity and opportunism of this species as has been described for other species of gulls e.g. [18, 42]. Studying Kelp Gull foraging strategies during other breeding stages might give a better overview of the range of foraging habitats used and the spatial requirements of this species in South Africa.

According to our prediction, colonies located further away from urban areas would feed more in natural habitats, but our results showed that some colonies located relatively far from urban centres (i.e. Malgas), spent more time in terrestrial anthropogenic areas (42%), than some colonies located within cities (i.e. Strandfontein in Cape Town). However, terrestrial anthropogenic habitats where birds from Malgas fed were mainly agricultural fields and artificial water bodies, whereas gulls from Strandfontein spent their time in highly degraded habitats such as the landfill or sewage plant both close to the colony. Even though gulls might use artificial water bodies and sewage plants for bathing or roosting, these habitats can potentially provide food in the forms of small fish or insects [63], while agricultural fields can offer food in the form of e.g. insects [64], annelids during ploughing [52], termite alates [65], or snails *Theba pisana* [27]. Therefore, Kelp Gulls in our study could feed to some extend on natural prey while foraging in anthropogenic habitats. It may be worth noting that gulls from the neighbouring colony, Jutten Island, located 3,7 km from Malgas, spent more time in the ocean and coastal areas, possibly to reduce intra-specific competition through spatial segregation [42, 66].

Finally, gulls from all our studied colonies spent a significant amount of time on the colony while incubating (75–87% of the tracking time), which was comparable to Ring-billed Gulls time budget (86.7% on colony; [67]), or Lesser Black-backed Gulls (75–80% on colony; [54]). The high colony attendance by Kelp Gulls might represent resting or feeding on the colony e.g. on insects as well as predating on other seabird eggs, or eggs and chicks from conspecifics (pers. obs; [68]), or kleptoparasiting other breeding seabirds [69]. It is likely that during food shortages, predation on conspecifics and other seabirds will increase with colony attendance, as foraging on or close to the colony can be beneficial for breeding success [58]. However, this situation will present a problem in mixed seabird colonies, such as Malgas and Jutten Island. Indeed, gulls from these colonies fed extensively in the marine environment and on terrestrial anthropogenic areas during our study and changes in the availability of these food sources could result in increased predation on other seabird eggs and chicks. For example, during the 2018 breeding season Kelp Gulls on Malgas predated on 8000 endangered Cape Gannet eggs [68], i.e. some 50% of the total gannet colony, which for a species that does not lay repeat clutches, will have serious population-level effects over the long term. The resolution of our GPS did not allow us to discriminate between time spend resting/ foraging on the colony or nest attendance, and it is possible that data from accelerometers may allow gain that insight by identifying behaviours such as standing, sitting or walking.

## Conclusions

This is the first comprehensive study using GPS loggers to investigate the foraging ecology of incubating Kelp Gulls in South Africa. We showed that like other *Larus* gulls, this opportunistic seabird is capable of foraging in various habitats, regardless of the proximity of their colony to urban areas or landfills. Additional information on Kelp Gull diet from stomach and pellet samples would be necessary to understand the energetic consequence of feeding in different habitats. Similarly, this study should be repeated during the chick-rearing stage to gain a more complete picture of the foraging ecology and energetics of South African Kelp Gulls. Such information is important to understand and predict the future population trajectory of Kelp Gulls in South Africa, with potential consequences on their environment and other species breeding in their vicinity.

As incubating Kelp Gulls in South Africa did not seem to depend highly on food made available from landfills, it is possible that changes in the availability of scraps due to improved landfill management (e.g. closing, covering, or diverting organic waste to composting facilities) might have little impact on South African Kelp Gull populations. The ability of Kelp Gulls in South Africa to exploit different foraging habitats allows this opportunistic forager to be highly adaptable and can thus be considered ‘winners’ of global change.

## Supplementary information

**Additional file 1.** 3D graph showing trip duration (min) and maximum distance (km) of foraging trips (%) between 0 and 1730 min and 0 and 90 km from colony in intervals.

**Additional file 2.** Overview of timing of data collection and number of adult Kelp Gulls deployed and recaptured per colony in South Africa in 2017 and 2018 with number of complete foraging trips performed.

**Additional file 3.** Time spent (%) in each of the foraging habitats in detail (Ocean, shore, natural, natural-transformed, urban, agriculture, landfill) for each of the six colonies.

## Data Availability

The datasets used and/or analysed during the current study are available from the corresponding author on reasonable request.

## Abbreviations

DW: Dwarskersbos
MA: Malgas Island
JU: Jutten Island
ST: Strandfontein
KE: Keurbooms Lookout Beach
SW: Swartkops Estuary
Km: Kilometers
M: Meters
Mm: Millimeters
H: Hours
Min: Minutes
G: Grams
EMbC: Expectation-Maximization Binary Clustering algorithm
LL: Low velocity and low turns
HL: High velocity and low turns
LH: Low velocity and high turns
HH: High velocity and high turns
A: Ocean
B: Coastal
C: Terrestrial natural
D: Terrestrial anthropogenic
