# Supplementary figures and images for: Foraging movements of breeding Kelp Gulls in South Africa
Source: Mov Ecol. 2020 Sep 3;8:36. doi: 10.1186/s40462-020-00221-x (PMC7469291; doi:10.1186/s40462-020-00221-x)

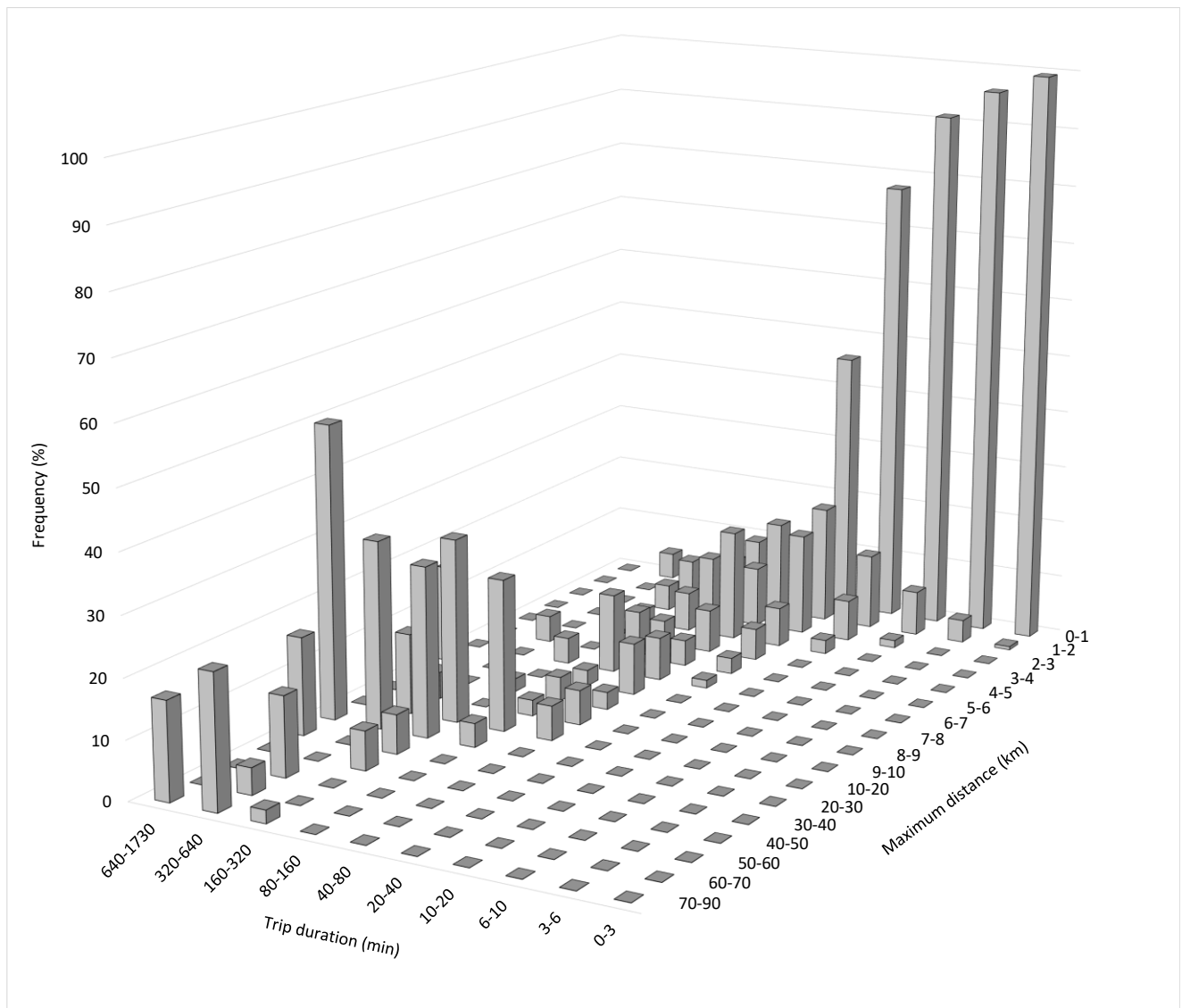

Supplement: Supplementary file 1 — Additional file 1. 3D graph showing trip duration (min) and maximum distance (km) of foraging trips (%) between 0 and 1730 min and 0 and 90 km from colony in intervals. [file 40462_2020_221_MOESM1_ESM.pdf]
