# Supplementary material for: Foraging movements of breeding Kelp Gulls in South Africa
Source: Mov Ecol. 2020 Sep 3;8:36. doi: 10.1186/s40462-020-00221-x (PMC7469291; doi:10.1186/s40462-020-00221-x)
Supplement: Supplementary file 2 — Additional file 2. Overview of timing of data collection and number of adult Kelp Gulls deployed and recaptured per colony in South Africa in 2017 and 2018 with number of complete foraging trips performed. [file 40462_2020_221_MOESM2_ESM.docx]

| Colony | Year tracked | Dates | Captured | Recaptured | Trips |
| --- | --- | --- | --- | --- | --- |
| Dwarskersbos | 2017 | 23/10-24/10 | 8 | 6 (75%) | 7 |
| Dwarskersbos | 2018 | 18/10-23/10 | 10 | 9 (90%) | 49 |
| Malgas Island | 2017 | 20/10-22/10 | 6 | 6 (100%) | 7 |
| Malgas Island | 2018 | 8/10-12/10 | 10 | 8 (80%) | 48 |
| Jutten Island | 2017 | 17/10-19/10 | 10 | 10 (100%) | 24 |
| Jutten Island | 2018 | 13/10-17/10 | 10 | 10 (100%) | 43 |
| Strandfontein | 2017 | 26/10-28/10 | 8 | 7 (87.5%) | 20 |
| Strandfontein | 2018 | 2/10-7/10 | 10 | 9 (90%) | 78 |
| Keurbooms | 2017 | 6/11-8/11 | 8 | 7 (87.5%) | 28 |
| Swartkops River | 2017 | 12/10-14/10 | 5 | 3 (60%) | 12 |
| **Total** |  |  | **85** | **75** | **316** |
