# Supplementary material for: Foraging movements of breeding Kelp Gulls in South Africa
Source: Mov Ecol. 2020 Sep 3;8:36. doi: 10.1186/s40462-020-00221-x (PMC7469291; doi:10.1186/s40462-020-00221-x)
Supplement: Supplementary file 3 — Additional file 3. Time spent (%) in each of the foraging habitats in detail (Ocean, shore, natural, natural-transformed, urban, agriculture, landfill) for each of the six colonies. [file 40462_2020_221_MOESM3_ESM.docx]

|  | Habitat | | | | | | |
| --- | --- | --- | --- | --- | --- | --- | --- |
| Colony | Ocean | Coastal | Natural | Natural-transf | Urban | Agriculture | Landfill |
| Dwarskersbos | 36.8% | 30.3% | 16.0% | 11.2% | 3.2% | 2.2% | 0.3% |
| Jutten Island | 51.7% | 12.8% | 11.7% | 11.6% | 2.8% | 3.5% | 5.8% |
| Malgas Island | 42.5% | 6.1% | 9.9% | 19.0% | 5.6% | 15.5% | 1.4% |
| Strandfontein | 20.6% | 21.6% | 24.3% | 14.7% | 3.4% | 0.3% | 15.0% |
| Keurbooms | 30.3% | 56.6% | 9.2% | 1.0% | 2.8% | 0% | 0% |
| Swartkops | 0% | 29.6% | 64.8% | 1.5% | 0.5% | 0% | 3.5% |
